# Cardiac progenitor cell-derived exosomes prevent cardiomyocytes apoptosis through exosomal miR-21 by targeting PDCD4

**DOI:** 10.1038/cddis.2016.181

**Published:** 2016-06-23

**Authors:** J Xiao, Y Pan, X H Li, X Y Yang, Y L Feng, H H Tan, L Jiang, J Feng, X Y Yu

**Affiliations:** 1 School of Pharmaceutical Sciences, Southern Medical University, Guangzhou, China; 2 Guangdong General Hospital, Guangdong Academy of Medical Sciences, Guangzhou, China; 3 School of Pharmaceutical Sciences, Guangzhou Medical University, Guangzhou, China

## Abstract

Cardiac progenitor cells derived from adult heart have emerged as one of the most promising stem cell types for cardiac protection and repair. Exosomes are known to mediate cell–cell communication by transporting cell-derived proteins and nucleic acids, including various microRNAs (miRNAs). Here we investigated the cardiac progenitor cell (CPC)-derived exosomal miRNAs on protecting myocardium under oxidative stress. Sca1^+^CPCs-derived exosomes were purified from conditional medium, and identified by nanoparticle trafficking analysis (NTA), transmission electron microscopy and western blotting using CD63, CD9 and Alix as markers. Exosomes production was measured by NTA, the result showed that oxidative stress-induced CPCs secrete more exosomes compared with normal condition. Although six apoptosis-related miRNAs could be detected in two different treatment-derived exosomes, only miR-21 was significantly upregulated in oxidative stress-induced exosomes compared with normal exosomes. The same oxidative stress could cause low miR-21 and high cleaved caspase-3 expression in H9C2 cardiac cells. But the cleaved caspase-3 was significantly decreased when miR-21 was overexpressed by transfecting miR-21 mimic. Furthermore, miR-21 mimic or inhibitor transfection and luciferase activity assay confirmed that programmed cell death 4 (PDCD4) was a target gene of miR-21, and miR-21/PDCD4 axis has an important role in anti-apoptotic effect of H9C2 cell. Western blotting and Annexin V/PI results demonstrated that exosomes pre-treated H9C2 exhibited increased miR-21 whereas decreased PDCD4, and had more resistant potential to the apoptosis induced by the oxidative stress, compared with non-treated cells. These findings revealed that CPC-derived exosomal miR-21 had an inhibiting role in the apoptosis pathway through downregulating PDCD4. Restored miR-21/PDCD4 pathway using CPC-derived exosomes could protect myocardial cells against oxidative stress-related apoptosis. Therefore, exosomes could be used as a new therapeutic vehicle for ischemic cardiac disease.

Cardiovascular disease is one of the leading pathological causes of mortality worldwide. Cardiovascular ischemic diseases such as heart failure, acute myocardial infarction and myocardial ischemia/reperfusion injury produce plenty of reactive oxygen species (ROS) in ischemic zone,^1, 2^ which is a major contributor to cardiomyocyte apoptosis and death, and deteriorates cardiac disease. Therefore, it is urgent to find an effective way to restore the cardiovascular system under oxidative stress.

Stem cell transplantation is an effective way to replace the apoptotic or dead cardiomyocytes, but the underlying mechanism of this repair process has not been fully explained. Cardiac progenitor cells (CPCs) resident in adult heart have emerged as one of the most promising stem cell types for cardiac regeneration and repair. The mechanism of post transplantation has always been predicated on the hypothesis that these cells would engraft, differentiate and replace damaged cardiac tissues. Although both direct cell differentiation and indirect paracrine effect mechanisms have been implicated in the therapeutic benefit, accumulating evidence suggests predominant roles of the paracrine secretion by CPCs.^3^ Furthermore, many researchers indicate that transplanted CPCs secrete a lot of factors to reduce tissue injury and/or enhance tissue repair.^4, 5^

Over the past few years, several experimental evidences have demonstrated that the CPCs released a specialized membranous nano-sized vesicle termed exosomes to improve cardiac function in the damaged heart.^5, 6, 7^ Exosomes are small (30–100 nm) membrane vesicles, merging their membrane contents into the recipient cell membrane and delivering effectors including transcription factors, oncogenes, small and large non-coding regulatory RNAs (such as microRNAs (miRNAs)), mRNAs and infectious particles into recipient cells.^8, 9^ In this way, exosomes secreted by CPCs were considered to participate in cardiac protection and repair.^7, 10, 11^ But exosomes contents vary from different pathological conditions, the difference might cause completely reversed fate of target cells. Hence, it is fruitful to investigate the biological function of exosomes under a specific pathological condition, including oxidative stress. In addition, this study will provide new theoretical basis for treatment of myocardium injury.

Among the contents of exosomes, miRNAs have been shown to govern important processes that contribute to the pathophysiological consequences of acute myocardial infarction.^12^ It is a class of short (about 22 nucleotides), single-stranded non-coding RNAs that have key roles in the regulation of gene expression. miRNAs can either promote or inhibit cardiomyocyte cell apoptosis,^13^ and also regulate ROS-mediated heart disease.^14, 15^ But whether miRNAs from CPC-derived exosomes have some important role in ROS-induced cardiomyocytes was still undetermined. Here we investigated the protective effect of the CPC-derived exosomes for myocardial cells in ischemic myocardial injury model, which is mainly through passing on exosomal miR-21 inhibit programmed cell death 4 (PDCD4) in myocardial cells. The fruitful work provides a potential cell therapy strategy for myocardial ischemic diseases.

## Results

### CPC-derived exosomes were collected and identified in morphology and phenotype

**Figure 1:**
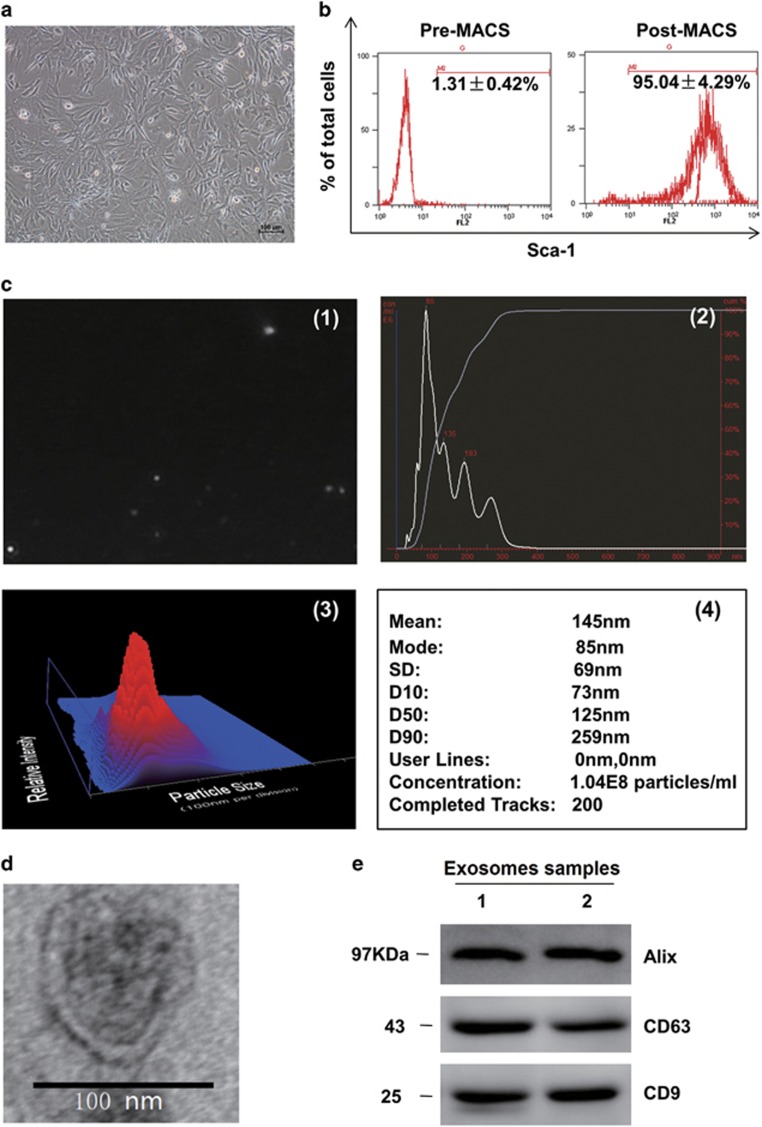
Characteristics of cardiac progenitor cells and exosomes. (**a**) The phase morphology of isolated CPCs growing on gelatin-coated dish; scale bar, 100 *μ*m. (**b**) Flow Cytometry analyzed purified Sca-1^+^CPCs from the first preparation. Typical purity of isolation is >95% after magnetic beads sorting. (**c**) Nanoparticle trafficking analyzed the diameters and concentration of exosomes; 1 is a representative screen shot of the NTA videos, the bright white dot indicates one moving particle, (2) NTA estimated the size of the EVs between 90 and 300 nm, and the mode of these particles is 85 nm, and predict the proper concentration is around 1.04 × 10^8^ particles per ml, the dilution is 1 : 80, 3 is a heat map pattern of 2, and 4 is a detail statistical report. (**d**) Electron micrograph analyzed CPC-derived exosomes. The image showed small vesicles of approximately 100 nm in diameter. Scale bar, 100 nm. (**e**) Western blotting characterized CPC-exosomes. CPC-exosomes preparation was separated by SDS-polyacrylamide gel electrophoresis, and electroblotted to the poly vinylidene fluoride (PVDF) membrane, and probed with exosomes marker CD63, CD9, Alix

The Sca-1^+^ cells isolated from adult mouse heart presented as long spindle-shaped fibrocyte-like adherent cells (Figure 1a). The percentage of Sca-1^+^ CPCs was determined with Flow Cytometry, and the results showed that up to 95.04±4.29% of population were Sca-1^+^ cells after magnetic-activated cell sorting (MACS; Figure 1b). To obtain the CPC-derived exosomes particles, the culture medium of CPCs was collected and precipitated. Then the morphology and phenotypes of isolated particles were identified according to the characteristics of exosomes described previously.^16^ First, the concentration and the range of size of the particles were measured using nanoparticle tracking analysis (Nanosight, Malvern, UK), the results demonstrated that the concentration of the particles was 1.31 × 10^9^±0.29 × 10^9^ particles per ml, and the diameters of the particles were within the range of 50–150 nm, with the average of 145 nm (Figure 1c). Secondly, the morphology of the CPC-derived particles was observed directly through transmission electron microscope (TEM), the particles were revealed as round-shaped vesicles with double layer membrane structure and diameters about 100 nm (Figure 1d). Finally, the protein levels of exosomes markers CD63, CD9 and Alix were measured with western blotting, all of the three markers could be detected in the CPC-derived exosomes (Figure 1e). So, the above properties analysis indicated that CPC-derived particles collected in our experiments were identified as exosomes.

### Oxidative stress enhanced the production of CPC-derived exosomes and caused apoptosis of cardiomyocytes

**Figure 2:**
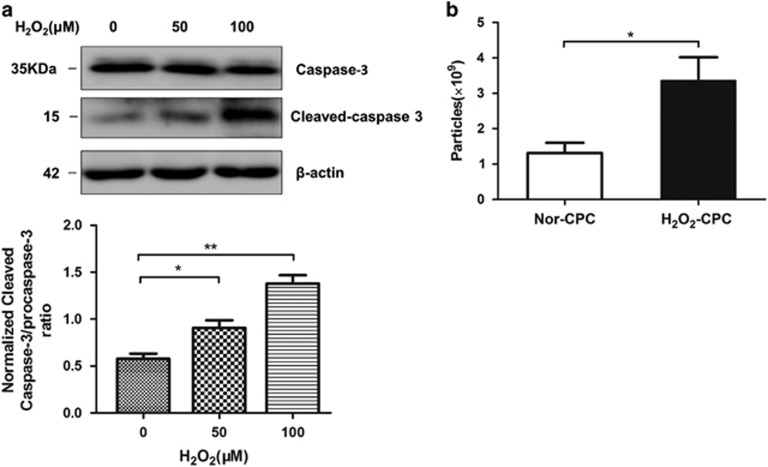
H_2_O_2_ affects the cells' apoptosis and exosomes' production. (**a**) Western blotting analyzed procaspase-3 and cleaved caspase-3 in H9C2 cells after treatment of different H_2_O_2_ concentrations (*N*=3, *P<0.05, **P<0.01 *versus* 0 *μ*M). (**b**) NTA counted the production of exosomes derived from normal cultured CPCs (Nor-exo) and exosomes from 100 *μ*M H_2_O_2_ treated CPCs (H_2_O_2_-exo), H_2_O_2_-CPC produce more exosomes ((3.36±0.66) × 10^9^ *versus* (1.31±0.29) × 10^9^, *N*=5; *P<0.05 *versus* Nor-CPC)

To test the effects of oxidative stress on cardiac cells, CPCs and H9C2 (a cardiomyocyte cell line) were treated with H_2_O_2_. After treated with H_2_O_2_ on indicated concentrations for 6 h, the H9C2 cells were harvested for protein collection and western blotting. The results showed that 100 *μ*M H_2_O_2_ increased the level of cleaved caspase-3 (the active type of caspase-3), suggesting an early apoptosis of the cardiomyocytes was induced under the oxidative stress (Figure 2a). Then, to observe whether the same condition of oxidative stress could affect the exosomes secretion of CPCs, exosomes were collected from CPCs treated with 100 *μ*M H_2_O_2_ for 6 h, and exosomes' concentration was analyzed with nanoparticle trafficking analysis (NTA). The results showed that the exosomes concentrations increased from 1.31±0.29 × 10^9^ particles per ml to 3.36±0.66 × 10^9^ particles per ml after the H_2_O_2_ treatment, suggesting that the oxidative stress could enhance the exosomes production of CPCs (Figure 2b).

### MiR-21 in the CPC-derived exosomes increased under the oxidative stress, potentially was involved in protecting cardiomyocytes from apoptosis

**Figure 3:**
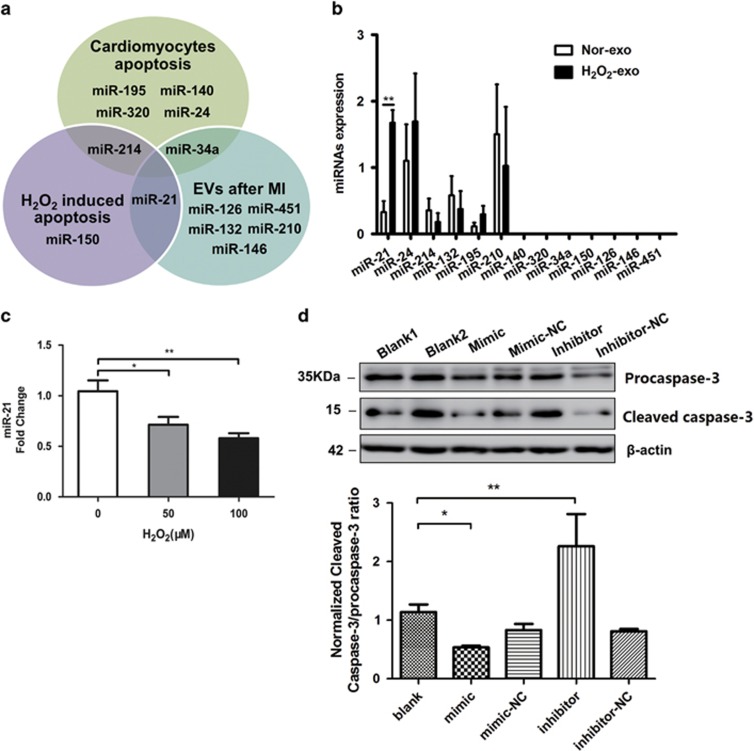
H_2_O_2_ changes the levels of miRNAs in CPC-exosomes. (**a**) Main reported miRNAs participated in cardiomyocytes apoptosis, H_2_O_2_ induced apoptosis and miRNAs contained in the EVs after myocardial infarction, some miRNAs are multifunctional in both fields, such as miR-21, miR-214 and miR-34a. (**b**) Quantitative PCR analysis of main reported miRNAs in Nor-exo and H_2_O_2-_exo, six miRNAs (miR-21, 24, 214, 132, 195, 210) are founded in H_2_O_2_-exo, and only miR-21 was significantly upregulated (*N*=6, **P<0.01 *versus* Nor-exo). (**c**) RT-qPCR analyzed and miR-21 in H9C2 cells after different concentrations of H_2_O_2_ treated (*N*=3, *P<0.05, **P<0.01 *versus* 0 *μ*M). (**d**) 100 *μ*M H_2_O_2_-treated H9C2 cells transfected with miR-21 mimics, inhibitors and both negative controls, and western blotting analyzed procaspase-3 and cleaved caspase-3 proteins levels in H9C2 cells (*N*=5, **P<0.01, ***P<0.001 *versus* blank group)

Exosome, acting as a carrier particle, has an intriguing role on cellular communication through the exchange of miRNAs or proteins between cells.^17^ It is essential to investigate the miRNAs contents with potential biological functions in exosomes secreted under certain pathological situations.^12^ So, we selected 13 miRNAs reported either involved in oxidative stress (miR-150, miR-21),^15, 18^ or cardiomyocytes apoptosis (miR-195, 320, 140, 24, 214, 34a),^19, 20, 21, 22^ or contained in extracellular vesicles (EVs) (miR-126, 146, 132, 210, 21, 451),^5, 23, 24, 25^ as shown in Figure 3a. Whether the oxidative stress would affect the profiles of these 13 miRNAs in CPC-derived exosomes was estimated through quantitative PCR. The agarose gel electrophoresis results showed that the cells and exosomal RNAs were integrated (Supplementary Figure S1). There were six miRNAs (miR-21, 24, 214, 132, 195, 210) detected in both H_2_O_2_-induced exosomes and non-induced ones. Among these miRNAs, miR-21 in CPC-derived exosomes was significantly upregulated (>5-fold change) after the H_2_O_2_ treatment (Figure 3b). This provided us a potential exosomal miRNA target, which might have a role on affecting the apoptosis of cardiomyocytes under the condition of the oxidative stress. The level of miR-21 was examined in H_2_O_2_-treated H9C2 cells, and the results showed that miR-21 were significantly downregulated in H9C2 cells under H_2_O_2_ treatment (Figure 3c), suggesting that a possible connection exists between the decrease of miR-21 and the apoptosis of cardiomyocytes with oxidative stress. So the gain-of- and loss-of-function experiments were performed using the mimic/inhibitor of miR-21. MiR-21 mimic or inhibitor obviously increase or decrease miR-21 expression in H9C2 specifically (Supplementary Figure S2). To detect the effects of miR-21 on the H_2_O_2_-induced apoptosis of cardiomyocytes, the levels of procaspase-3 and cleaved caspase-3 were detected by western blotting. The results showed that miR-21 mimic obviously decreased cleaved caspase-3 expression, whereas the inhibitor increased cleaved caspase-3 expression (Figure 3d) in H9C2 under oxidative stress (100 *μ*M H_2_O_2_). This confirmed the anti-apoptotic function of miR-21 and implied that rescuing the downregulated miR-21 in the cardiomyocytes under oxidative stress might be a potential strategy to protect the cardiomyocytes from apoptosis.^18, 26^

### MiR-21 inhibited the apoptosis of H9C2 by targeting PDCD4

**Figure 4:**
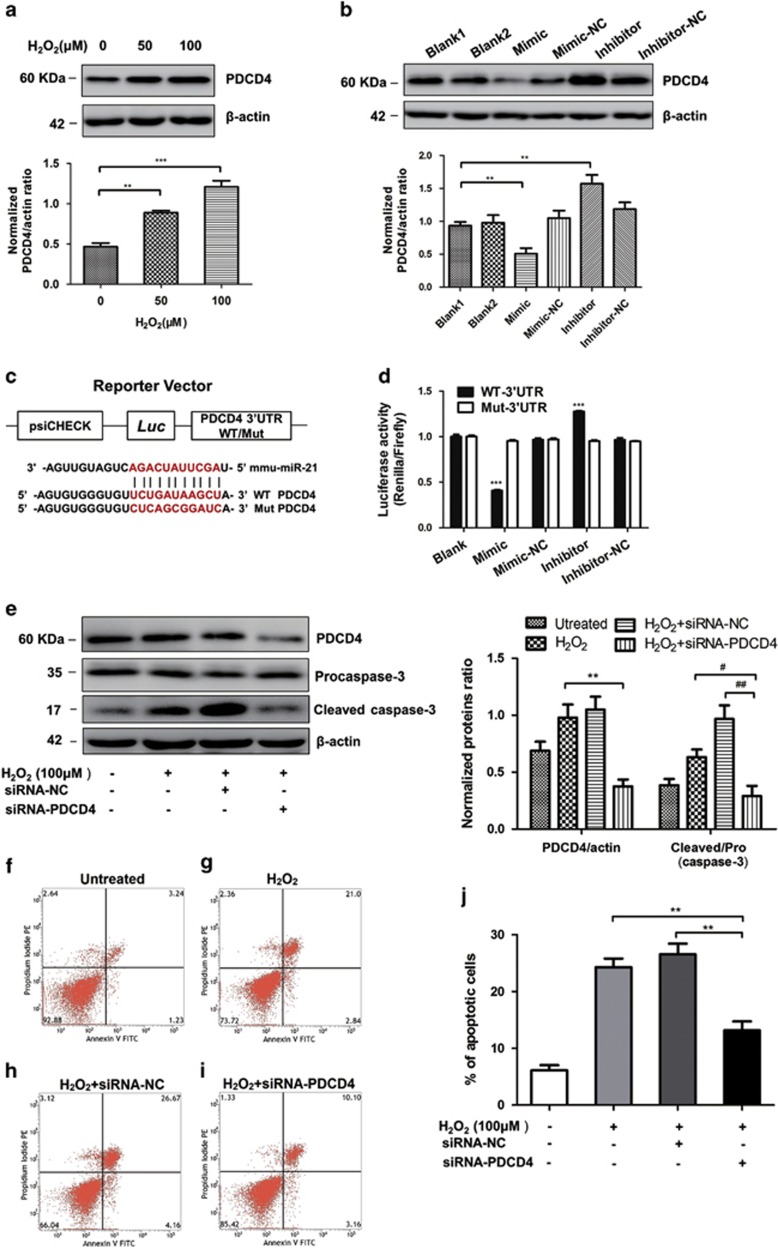
PDCD4 is a target gene of miR-21. (**a**) Western blotting analyzed PDCD4 in H9C2 cells after different concentrations of H_2_O_2_ treated (*N*=4, **P<0.01, ***P<0.001 *versus* 0 *μ*M). (**b**) One hundred micromolar H_2_O_2_-treated H9C2 cells transfected with miR-21 mimics, inhibitors and both negative controls, western blotting analyzed PDCD4 protein levels in H9C2 cells (*N*=3, *P<0.05, **P<0.01, ***P<0.001 *versus* blank group). (**c**) Schematic representation of 3′-UTR of PDCD4 mRNA reporter with and without the miR-21 seed-binding site (red). (**d**) Luciferase activity assay of HEK293T cells transfected with luciferase constructs containing WT-3′UTR and Mut-3′UTR of PDCD4 (*N*=3, ***P<0.001 *versus* blank group). (**e**) Western blot analyzed the protein levels of caspase-3 and PDCD4 after H9C2 transfected with siRNA-PDCD4 (*N*=3, **P<0.01 *versus* H_2_O_2_ group, ^#^*P*<0.05, ^##^*P*<0.01 *versus* siRNA-PDCD4 group). (**f**–**h**) Representative dot plots of cell apoptosis were showed after Annexin V/PI dual staining. The proportion of dead cells (Annexin V−/PI+), live cells (Annexin V−/PI−), early apoptotic cells (Annexin V+/PI−) and late apoptotic/necrotic cells (Annexin V+/PI+) was respectively measured for comparison. (**j**)The percentage of apoptotic cells was represent for both early and late apoptotic cells (*N*=3, **P<0.01 *versus* siRNA-PDCD4 group)

PDCD4, a promoter of tumor cell apoptosis and suppressor of tumor metastasis, as a predicted target gene of miR-21 in tumor cells.^27^ As shown in Supplementary Figure S3, there are conserved binding sites in 3′UTR of PDCD4 mRNA in different species. To confirm whether PDCD4 is a target of miR-21 in H9C2 cells, the expression levels of PDCD4 in H_2_O_2_-treated H9C2 cells were measured by western blotting. The results showed that PDCD4 was significantly upregulated in H9C2 cells under H_2_O_2_ treatment (Figure 4a). Furthermore, gain-of- and loss-of-function assays showed that miR-21 inhibitor increased, whereas miR-21 mimic decreased PDCD4 mRNA (Supplementary Figure S4) and protein (Figure 4b) levels in cardiomyocytes. This data indicated that miR-21 possibly attenuated cell apoptosis through inhibiting the expression of PDCD4.

To further address whether miR-21 directly binds the 3′UTR region of PDCD4, we generated some chimeric constructs which harbor luciferase wild-type 3′UTR sequence (WT-3′UTR) or mutant 3′UTR sequence (Mut-3′UTR; Figure 4c). As expected, miR-21 mimic exclusively inhibited the luciferase activity of Luci-WT-3′UTR, suggesting that the putative binding site is important for miR-21 suppressing PDCD4 expression (Figure 4d). We next want to explore the relationship between PDCD4 and apoptosis in H9C2 cells. Through siRNA-mediated gene silence, we found that PDCD4-siRNA apparently decreased the cleaved caspase-3 level, (Figure 4e). The knockdown efficiency of PDCD4-siRNA was also detected by western blotting (Figure 4e, upper). In addition, the Annexin V/PI assay showed that PDCD4 downregulated cells decreased the percentage of the apoptotic cells to 13.26%, compared with the 23.84% in H_2_O_2_ group and 30.83% in siRNA-NC group (Figure 4f and j). Taken together, these results confirmed that the effects of miR-21 on oxidative stress-induced apoptosis were through targeting PDCD4.

### CPC-derived exosomes restored the miR-21-PDCD4 pathway and attenuated apoptosis in cardiomyocytes under oxidative stress

**Figure 5:**
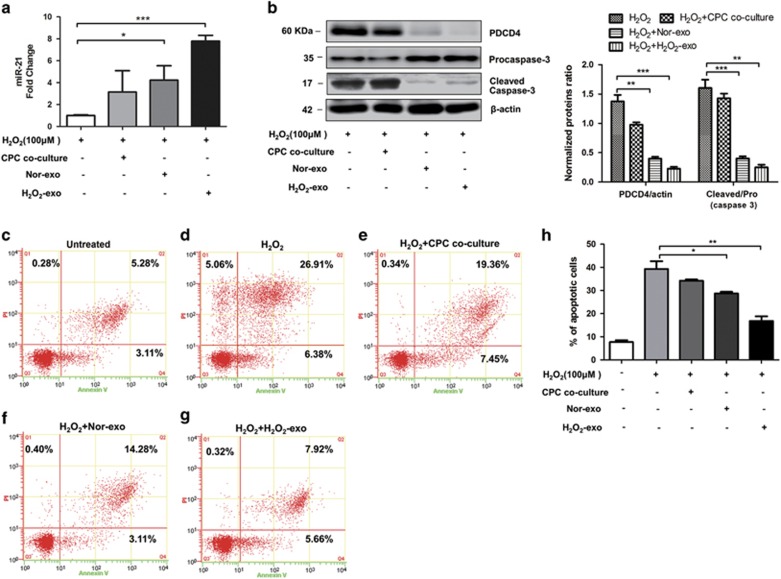
CPC-exosomes inhibit the apoptotic function caused by H_2_O_2_ in cardiomyocytes. (**a**) RT-qPCR analyzed miR-21 in H9C2 cells under 100 *μ*M H_2_O_2_ after CPCs co-culture, Nor-exo and H_2_O_2_-exo pre-protection (*N*=4, *P<0.05, ***P<0.001 *versus* control group). (**b**) Western blotting analyzed procaspase-3, cleaved caspase-3 and PDCD4 in H9C2 cells under 100 *μ*M H_2_O_2_ after CPCs co-culture, Nor-exo and H_2_O_2_-exo pre-protection (*N*=4, **P<0.01, ***P<0.001 *versus* control groups). (**c**–**g**) Representative dot plots of cell apoptosis were showed after Annexin V/PI dual staining. (**h**) The percentage of apoptotic cells was represent for both early and late apoptotic cells (*N*=3, *P<0.05, **P<0.01 *versus* H_2_O_2_ groups)

Exosomes are important media to regulate cell-to-cell communication. The first step for exosomes releasing their cargoes into target cells is through fusing themselves into the membrane of target cells. To determine whether CPC-exosomes can be taken up by cardiomyocytes, CPC-exosomes were labeled with PKH26, a fluorescent cell linker compound that is incorporated into the cell membrane by selective partitioning. After incubating the labeled exosomes with cardiomyocytes for 12 h, the exosomes pellet show strong red fluorescence in the cytoplasm of H9C2 cells (Supplementary Figure S5), indicating that lots of exosomes were taken up by the H9C2 cells. Not surprisingly, when the cells were pre-treated with CPC-derived exosomes, the decrease of miR-21 was rescued (Figure 5a) under oxidative stress and the increases of PDCD4 and cleaved caspase-3 were suppressed (Figure 5b). Consistent with the higher yields of exosomes and exosomal miR-21 under oxidative stress, the exosomes derived from H_2_O_2_-treated CPCs showed even stronger effects on increasing miR-21 levels and decreasing PDCD4 expression in the receptor cells. Whether the CPC-derived exosomes protected cardiomyocytes from the apoptosis caused by oxidative stress, an Annexin V/PI analysis was carried out. The results showed that the cells pre-treated with H_2_O_2_-exosomes decreased the percentage of the apoptotic cells to 13.58%, compared with the 33.29% in H_2_O_2_ group, whereas the normal exosomes (non-H_2_O_2_ induced) could only reduce the apoptotic percentage to 17.39% (Figure 5c–h). Therefore, the CPC-derived exosomes might be crucial to protect the cardiomyocytes from apoptosis caused by oxidative stress, and this effect was achieved by delivering miR-21 to targeting PDCD4 in the receptor cardiomyocytes.

## Discussion

Oxidative stress has been identified as critical in many key steps in cardiac diseases, such as atrial enlargement,^28^ mitral regurgitation^29^ and heart failure.^30^ Stem cell–based therapies have shown promise to repair detrimental myocardial remodeling and cardiac dysfunction, but significant obstacles to this approach remain. Thus, the amplification and delivery of beneficial paracrine signals generated by stem cells could overcome obstacles associated with cell injection–based approaches to repair damaged myocardium.^31^ Because CPCs are specialized to function in the heart, CPC-generated signals may be particularly well suited to treat cardiac pathologies,^32^ the paracrine effect of CPCs has considered to be an important mechanism of cardiac protection.^7^ Exosomes, one of the most important paracrine factors, has been reported in many other cells, such as tumor cells and stem cells, the cargoes of exosomes are verified as the crucial signaling molecular for some key pathway.^9, 33^ Very few studies have focused on the anti-oxidative stress potential of CPC-derived exosomes. In the present study, we obtained the round, double layer, had a diameter of 30–100 nm vesicles from CPCs conditioning medium, and express specific protein markers of exosomes.

The oxidative stress originates mainly in mitochondria from ROS and can be identified in the pathophysiology of the consequential clinical manifestations of cardiovascular disease.^34^ In fact, transplanted CPCs and resident cardiomyocytes are stayed in the same oxidative environment. It is important to analyze exosomes induced by oxidative stress, similar to the pathological state when transplanted CPCs embedded in pathological area. In the present study, we use H_2_O_2_ induce the oxidative stress to mimic the microenvironment of cardiomyocytes and CPCs under some certain cardiovascular disease. The results demonstrated that H_2_O_2_ effectively induced cardiomyocytes apoptosis, and the same concentration of H_2_O_2_ not only elevate the generation of exosomes from conditioned culture medium of CPCs, but also change exosomal miRNA levels. Previous research found that pathological microenvironment or cell species could not only change the production of exosomes, but also the cargoes in exosomes, including miRNAs,^6, 11, 35^ proteins^36, 37^ and lncRNA.^10, 38^ Chen *et al.*^4^ reported that miR-451 contained in CPC-derived exosomes had cardioprotection roles for acute ischemia/reperfusion injury. However, we did not observe the obvious expression of miR-451 with qPCR in our experimental system, either in normal exosomes or in H_2_O_2_ induced ones. Considered that miRNAs normally express and act in a very sensitive manner, we propose the explanation to the differences of the exosomal miRNAs described in our and others work is due to the different experiment conditions, including different concentration or treated time of stimulus. However except the certain miRNAs, other molecules were not evaluated in this study.

miRNAs are small non-coding RNAs that block translation or induce degradation of mRNA and thereby control patterns of gene expression.^39^ Many miRNAs have reported to contribute to the pathophysiological consequences of acute myocardial infarction.^12^ Several miRNAs regulate apoptosis and survival pathways in cardiomyocytes, inhibition of apoptosis or activation of survival programs enhances cardiac regeneration. Proapoptotic miRNAs include the miR-15 family,^20^ miR-34,^19^ miR-320,^40^ and miR-140,^22^ and anti-apoptotic miRNAs include miR-24,^41^ miR-214,^13, 21^ miR-145,^14^ miR-150^15^ and miR-21.^42^ Some of them could regulate H_2_O_2_ induced cell apoptosis, like miR-150,^15^ miR-21^42^ and miR-103/107.^43^ Other miRNAs, which found in extracellular vesicles or exosomes, are reported to have an essential role in cardiac regeneration,^24^ including miR–126,^25^ miR–132,^24^ miR–146^44^ and miR–210.^5^ Here we detected six miRNAs of 12 were encapsulated by CPC-exosomes under oxidative stress. Interestingly, we found only the miR-21 upregulated by ≥5-fold by oxidative stress at the 6-h time point, probably to be involved in regulating cardiac functions of interest. miRNAs normalized by U6, U6 as a housekeeping gene was widely used in the miRNAs quantification, including exosomal miRNAs quantification, but Lin *et al.*^45^ found that human U6 promoter activity was downregulated in the presence of hydrogen peroxide. In our study, we use hydrogen peroxide to induce oxidative stress for cardiomyocytes, and our study mainly focused on the exosomal miRNAs, which respond to environmental stimulus, in a different profile than cellular miRNAs do. When we compared the expression of exosomal U6 from oxidative stress-treated cells to the one of untreated control cells, there were no noticeable differences. The research about U6 regulated by oxidative stress was mostly focused on the human U6 promoter, whereas our work was focused on rat cardiomyocytes (H9C2). Meanwhile, there was few research demonstrated that U6 expression could be influenced by oxidative stress in rat. As the regulation of U6 could have species diversity, a detailed study is necessary to clarify this subject.

miR-21 has been reported to mediate gene regulation and cellular injury response in H_2_O_2_-induced vascular smooth muscle cells.^18^ Our results demonstrated that oxidative stress caused decrease of miR-21 levels in cardiomyocytes, indicated that miR-21 may be one of the key factors to regulate cardiomyocytes function under oxidative stress. In the gain-of- and loss-of-function experiments, we found that upregulated miR-21 levels effectively inhibit H_2_O_2_-induced cardiomyocytes apoptosis. Usually miRNAs mediate cell function by inhibiting the post-transcription process of downstream target genes; previous studies revealed that miR-21 specifically targets and regulates PDCD4, PTEN, RECK and Bcl-2 in tumor proliferation, invasion and migration.^26, 27, 46^ Our data confirmed that PDCD4 was negatively regulated by miR-21 in oxidative stress-induced cardiomyocytes, and PDCD4 silencing inhibited the anti-apoptosis function of miR-21. These findings strongly supported that PDCD4 is the underlying mechanism of miR-21-mediated cellular protection.

Exosomes as a mediator to regulate cell-to-cell communication, such as stromal cells to breast cancer cells, mesenchymal stem cells to endothelial cells, has been fully proved.^47, 48^ The understanding of exosomes biogenesis and endocytosis was incomplete, whether exosomes could specifically recognize their receptor cells still needs to be deeply explored.^9^ In our study, when the cardiomyocytes were pre-treated with CPC-derived exosomes, the exosomes can be taken up at high efficiency, and the exosomal miR-21 also can be transferred into the cardiomyocytes and take part in the cellular signaling pathway. This delivery successfully caused downstream response, including the decreasing PDCD4 expression and percentage of apoptotic cells in the cardiomyocytes. So CPC-exosomes can rescue injured cardiomyocytes by reconstructing the miR-21/PDCD4 pathway, CPCs exosomal miR-21 may be one of the protective factors to prevent cardiomyocytes from apoptotic process. Although our data suggest that exosomal miR-21 had a critical role in the apoptotic regulation of recipient cells, we do not rule out the contribution of other exosomal cargoes. Whether CPC-exosomes have same function in animal heart disease models, the mechanism remains to be further explored.

**Figure 6:**
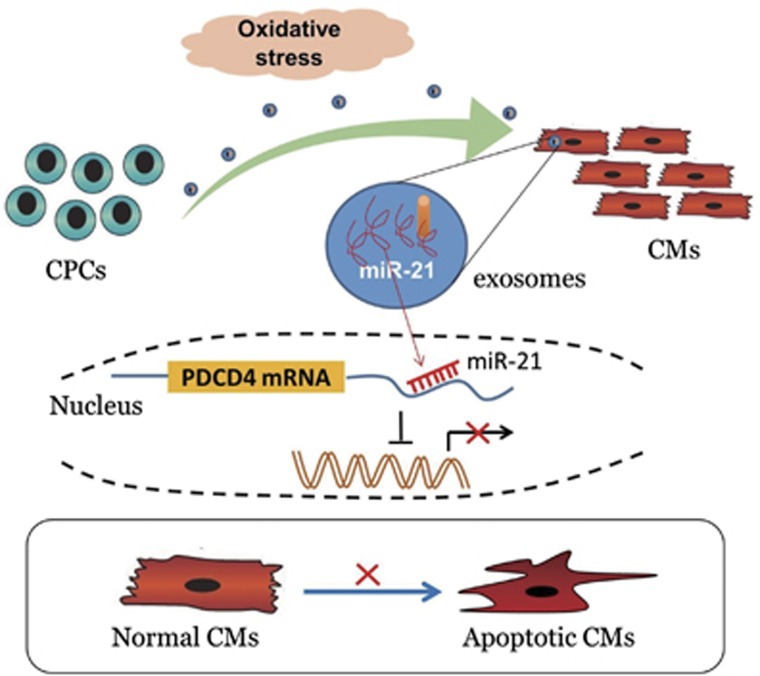
Schematic of our working hypothesis. In some oxidative stress-related cardiac injury, CPCs secreted a considerable amount of exosomes, which can be taken up by cardiomyocytes, and miR-21 contained in CPC-exosomes had an inhibiting role in the apoptosis pathway through downregulating PDCD4

In conclusion, the fruitful work gives explanation to an intricate exosome-mediated cross-talk of CPCs and cardiomyocytes (Figure 6). CPC-derived exosomes prevent cardiomyocytes apoptotic program, at least partly, via miR-21 contained in exosomes. Generally, our data indicate that individual species of miRNA have a crucial role in exosomes function. Therefore, we can get the conclusion that the exosomal miR-21 could be demonstrated as a promising therapeutic strategy for ROS-mediated cardiac disease.

## Materials and Methods

### Isolation of cardiac progenitor cells

Cardiac progenitor cells were generated from the hearts of 4-week-old, male, C57BL/6 mice (The Sun-yet sen University experimental animals center, Guangzhou, China) isolated by using MACS (Stem Cell Technology, Vancouver, BC, Canada). Briefly, cardiac tissues were minced into 1 mm^3^ explants then digested by 0.1% collagenase (Gibco, Waltham, MA, USA) into single cell suspension; next, Sca-1^+^ cells were isolated through MACS with Sca-1 magnetic beads under the manufacturers' instruction. The selected Sca-1^+^ cells were cultured and maintained in complete media containing M199 (Corning, Corning, NY, USA), EGM-2 (Lonza, Walkersville, MD, USA), 10% exosomes-depleted FBS (Exo-FBS, System Biosciences, Mountain View, CA, USA), 10 nM b-FGF, 1% MEM nonessential amino acids (Gibco, USA), and penicillin–streptomycin (Gibco, USA).

### Exosomes purification

The CPC-exosomes isolation procedures were performed as previously described.^49^ Briefly, 10 ml conditional culture medium with 10% Exo-FBS was used for culturing CPCs in T75 flask. After 48 h, supernatant was centrifuged at 3000 r.p.m., 15 min to remove cells, followed by filtration through 0.22 *μ*m filter to remove cell debris. Exosomes in medium were precipitated with ExoQuick TC (System Biosciences) under the manufacturer's instruction, then resuspended exosomes pellets in 50 *μ*l phosphate-buffered saline (PBS) and stored at –80 °C.

### Transmission electron microscopy

For the TEM morphology investigation, 3 *μ*l of exosomes pellet was placed on formvar carbon-coated 200-mesh copper electron microscopy grids, and incubated for 5 min at room temperature, and then was subjected to standard uranyl acetate staining. The grid was washed with three changes of PBS and allowed to semi-dry at room temperature before observation in transmission electron microscope (Hitachi H7500 TEM, Tokyo, Japan). Micrographs were used to quantify the diameter of exosomes.

### Nanoparticle trafficking analysis

Analysis of absolute size distribution of exosomes was performed using NanoSight NS300 (Malvern, UK). With NTA, particles are automatically tracked and sized based on Brownian motion and the diffusion coefficient. After isolation, the EVs were diluted in 1 ml of filtered PBS. Control medium and filtered PBS were used as controls. The NTA measurement conditions were temperature 23.75±0.5 °C, 25 frames per second, measurement time 60 s. The detection threshold was similar in all the samples. Three recordings were performed for each sample.

### Immunoblotting

Exosomes lysate supernatants were prepared, exosomes were assessed for their protein content using BCA Protein Assay Kit (Thermo, Waltham, MA, USA), and then resolved on a 10% sodium dodecyl sulfate bis-tris gel, and transferred to an Immobilon FL PVDF membrane (Millipore, Billerica, MA, USA). The membrane was blocked with 5% non-fat milk in TBST buffer, and incubated with rabbit anti-CD63 (1 : 500, SC-15363, Santa Cruz Biotechnology, Dallas, TX, USA), CD9 (1 : 1000, Abcam, Cambridge, MA, USA) and Alix (1 : 1000, Abcam) overnight, and then washed with TBST, continued to incubate with HRP linked goat anti-rabbit IgG (1 : 5000, Cell Signaling Technology, Danvers, MA, USA), and the protein bands were visualized using the automatic imager (General Electric, Fairfield, CT, USA).

### Isolation and quantification of miRNAs

Total RNA from exosomes were extracted by SeraMir (System Biosciences) following the manufacturer's instructions, cell RNAs extracted by RNAprep pure cell/bacteria Kit (TIANGEN, Beijing, China). The purity of isolated RNA was determined by OD260/280 using a Nanodrop ND-1000 (Thermo Scientific), and integrity assessed by agarose gel electrophoresis. Isolated RNAs were reverse transcription using the PrimeScipt RT Reagent Kit (TaKaRa, Kusatsu, Shiga, Japan). The cDNA was used to perform quantitative PCR on BioRad Real-Time PCR System (BioRad, Hercules, CA, USA) using the SYBR kit (BioRad, USA). Amplification was performed at 95 °C for 5 min, followed by 40 cycles of 95 °C for 10 s, 55.7 °C for 30 s. Quantification of miR-21a-5p, miR-24-3p, miR-195a-5p, miR-132, miR-214 and miR-210 and so on were performed with a stem-loop real-time PCR miRNA kit (Ribobio, Guangzhou, China). miRNA primer also subscribed from Ribobio company (Guangzhou, China). Fold-induction was calculated using the Ct method: ΔΔCt=(Ct_Target miRNA_−Ct_U6_) H_2_O_2_ induced exosomes—(Ct_Target miRNA_−Ct_U6_) normal exosomes, and the final data were derived from 2^−ΔΔCt^.

### H9C2 transfection

MiR-21 mimics, inhibitors and negative control RNAs (ribobio, Guangzhou, China) were transfected with lipofectamine2000 (Life Technologies) according to manufacturer's guidelines. The work concentration of mimics and mimic-NC were 50 nM, whereas inhibitors and inhibitor-NC were 100 nM. After 6 h, medium was refreshed. After 48 h, cells were harvested for total RNA and proteins extraction. The efficiency of mimics or inhibitors was confirmed by RT-qPCR. Following the PDCD4 mRNA, the PDCD4 and caspase-3 protein expression levels were detected by RT-qPCR and western blotting. Total RNA from cells were extracted by RNAprep pure cell/bacteria Kit (TIANGEN) following the manufacturer's instructions. Isolated RNAs were reverse transcripted using the FastQuant RT super mix Kit (TIANGEN). The cDNA was used to perform quantitative PCR on BioRAD Real-Time PCR System (BioRad) using the SYBR kit (BioRad). Amplification was performed at 95 °C for 5 min, followed by 40 cycles of 95 °C for 10 s, 60 °C for 20s, 72 °C 15 s. Fold-induction was calculated using the Ct method: ΔΔCt=(Ct_PDCD4_–Ct_actin_) treated cells–(Ct_PDCD4_–Ct_actin_) normal cells, and the final data were derived from 2^−ΔΔCt^. Whole-cell proteins were extracted for western blotting analysis, the procedures were same as above described.

### Luciferase reporter assay

HEK293 cells were co-transfected with 500 ng psiCHECK-2–PDCD4-3′UTR (wild type and mutant type) and 50 nM miR-21 mimic, 100 nM inhibitor and negative control (ribobio) using Lipofectamine2000 (Invitrogen, USA) following the manufacturer's instructions. After 48 h cells were lysed to Dual-Luciferase Reporter Assay System (Promega, Madison, WI, USA), and luciferase activity was measured using a GloMax20/20 Luminometer (Promega). Luciferase activity was normalized by Renilla/Firefly luciferase signal in HEK293 cells.

### RNA interference

The synthesized siPDCD4 and scramble (GeneCopoeia, Rockville, MD, USA) were transfected into cells with Lipofectamine (Invitrogen, Carlsbad, CA, USA), according to the manufacturer's instructions. Briefly, H9C2 cells (8 × 10^4^ per well) were plated onto 6-well plates and allowed to grow for 12 h. siPDCD4 (50 nM) or scramble with 5*μ*l lipofectamine were added into cells. After transfection, cells were incubated at 37 °C for 24 h to 72 h, then treated in the presence of 100 *μ*M H_2_O_2_ for 6 h. Cells were harvested for analysis after the indicated time points as described above, and the proteins were for western blot assay and cells for Annexin/PI assay.

The siPDCD4 sequence: F: GAAAGCGUAAGGAUAGUGUdTdT,

R: ACACUAUCCUUACGCUUUCdTdT

### Apoptosis assay of H9C2 treated with CPC-derived exosomes

H9C2 cells were cultured in DMEM/F12 (Hyclone, Logan, UT, USA) medium supplemented with 10% FBS, H9C2 cells were pre-incubated with 10% Exo-FBS DMEM with stressed, normal or without CPC-exosomes (2 × 10^9^ particles per ml) for 24 h, then treated with 100 *μ*M H_2_O_2_ for 6 h. Following treatment, the apoptosis rate analyzed by the Flow cytometry with Annexin V/PI kit (BD Bioscience, Franklin Lakes, NJ, USA), according to the instructions of the manufacturer. Whole-cell lysate were prepared by adding cell lysis buffer (Thermo), cell protein concentration determined by BCA Protein Assay Kit (Thermo), and then resolved on a 10% sodium dodecyl sulfate bis-tris gel, and transferred to an Immobilon FL PVDF membrane (Millipore). The membrane was blocked with 5% non-fat milk in TBST buffer, and incubated with rabbit anti-caspase-3 (1 : 1000, #9662, Cell Signaling Technology), rabbit anti-PDCD4(1 : 1000, #9535, Cell Signaling Technology) overnight, and then, incubated with HRP linked goat anti-rabbit IgG (1 : 5000, Cell Signaling Technology), and the protein bands were visualized using the with automatic imager (General Electric). The blots were quantified using FluorChem 8900 software (Alpha Innotech Corporation, San Leandro, CA, USA), and the relative protein expression was normalized to *β*-actin.

### Statistical analysis

An unpaired *t*-test and one-way ANOVA were performed using GraphPadPrism version 5.0 for Windows (GraphPad Software, Inc., La Jolla, CA, USA) to determine *P*-value in repeated experiments. All values are expressed as mean±S.E.M. A value of *P*<0.05 was considered to indicate statistically significant differences. Unless otherwise noted, all results were obtained through a minimum of three independent experimental replications.

## Abbreviations

CPCs: cardiac progenitor cells
miRNA: microRNA
PBS: phosphate-buffered saline
MACS: magnetic-activated cell sorting system
NTA: nanoparticle trafficking analysis
PDCD4: programmed cell death 4

## References

[bib1] Lefer DJ, Granger DN. Oxidative stress and cardiac disease. Am J Med 2000; 109: 315–323. 10996583 10.1016/s0002-9343(00)00467-8

[bib2] Cantor EJ, Mancini EV, Seth R, Yao XH, Netticadan T. Oxidative stress and heart disease: cardiac dysfunction, nutrition, and gene therapy. Curr Hypertens Rep 2003; 5: 215–220. 12724053 10.1007/s11906-003-0023-z

[bib3] Chimenti I, Smith RR, Li TS, Gerstenblith G, Messina E, Giacomello A et al. Relative roles of direct regeneration versus paracrine effects of human cardiosphere-derived cells transplanted into infarcted mice. Circ Res 2010; 106: 971–980. 20110532 10.1161/CIRCRESAHA.109.210682 PMC4317351

[bib4] Chen L, Wang Y, Pan Y, Zhang L, Shen C, Qin G et al. Cardiac progenitor-derived exosomes protect ischemic myocardium from acute ischemia/reperfusion injury. Biochem Biophys Res Commun 2013; 431: 566–571. 23318173 10.1016/j.bbrc.2013.01.015 PMC3732190

[bib5] Barile L, Lionetti V, Cervio E, Matteucci M, Gherghiceanu M, Popescu LM et al. Extracellular vesicles from human cardiac progenitor cells inhibit cardiomyocyte apoptosis and improve cardiac function after myocardial infarction. Cardiovasc Res 2014; 103: 530–541. 25016614 10.1093/cvr/cvu167

[bib6] Gray WD, French KM, Ghosh-Choudhary S, Maxwell JT, Brown ME, Platt MO et al. Identification of therapeutic covariant microRNA clusters in hypoxia-treated cardiac progenitor cell exosomes using systems biology. Circ Res 2015; 116: 255–263. 25344555 10.1161/CIRCRESAHA.116.304360 PMC4338016

[bib7] Sluijter JP, van Rooij E. Exosomal microRNA clusters are important for the therapeutic effect of cardiac progenitor cells. Circ Res 2015; 116: 219–221. 25593269 10.1161/CIRCRESAHA.114.305673

[bib8] Hu G, Yao H, Chaudhuri AD, Duan M, Yelamanchili SV, Wen H et al. Exosome-mediated shuttling of microRNA-29 regulates HIV Tat and morphine-mediated neuronal dysfunction. Cell Death Dis 2012; 3: e381. 22932723 10.1038/cddis.2012.114 PMC3434655

[bib9] EL Andaloussi S, Mager I, Breakefield XO, Wood MJ. Extracellular vesicles: biology and emerging therapeutic opportunities. Nat Rev Drug Discov 2013; 12: 347–357. 23584393 10.1038/nrd3978

[bib10] Mohankumar S, Patel T. Extracellular vesicle long noncoding RNA as potential biomarkers of liver cancer. Brief Funct Genomics 2015; 15: 249–256. 26634812 10.1093/bfgp/elv058 PMC4880007

[bib11] Valadi H, Ekstrom K, Bossios A, Sjostrand M, Lee JJ, Lotvall JO. Exosome-mediated transfer of mRNAs and microRNAs is a novel mechanism of genetic exchange between cells. Nat Cell Biol 2007; 9: 654–659. 17486113 10.1038/ncb1596

[bib12] Boon RA, Dimmeler S. MicroRNAs in myocardial infarction. Nat Rev Cardiol 2015; 12: 135–142. 25511085 10.1038/nrcardio.2014.207

[bib13] Lv G, Shao S, Dong H, Bian X, Yang X, Dong S. MicroRNA-214 protects cardiac myocytes against H2O2-induced injury. J Cell Biochem 2014; 115: 93–101. 23904244 10.1002/jcb.24636

[bib14] Cha MJ, Jang JK, Ham O, Song BW, Lee SY, Lee CY et al. MicroRNA-145 suppresses ROS-induced Ca2+ overload of cardiomyocytes by targeting CaMKIIdelta. Biochem Biophys Res Commun 2013; 435: 720–726. 23702479 10.1016/j.bbrc.2013.05.050

[bib15] Li X, Kong M, Jiang D, Qian J, Duan Q, Dong A. MicroRNA-150 aggravates H2O2-induced cardiac myocyte injury by down-regulating c-myb gene. Acta Biochim Biophys Sin (Shanghai) 2013; 45: 734–741. 23824072 10.1093/abbs/gmt067

[bib16] Cosme J, Liu PP, Gramolini AO. The cardiovascular exosome: current perspectives and potential. Proteomics 2013; 13: 1654–1659. 23526783 10.1002/pmic.201200441

[bib17] Braicu C, Tomuleasa C, Monroig P, Cucuianu A, Berindan-Neagoe I, Calin GA. Exosomes as divine messengers: are they the Hermes of modern molecular oncology? Cell Death Differ 2015; 22: 34–45. 25236394 10.1038/cdd.2014.130 PMC4262777

[bib18] Lin Y, Liu X, Cheng Y, Yang J, Huo Y, Zhang C. Involvement of MicroRNAs in hydrogen peroxide-mediated gene regulation and cellular injury response in vascular smooth muscle cells. J Biol Chem 2009; 284: 7903–7913. 19158092 10.1074/jbc.M806920200 PMC2658083

[bib19] Bernardo BC, Gao XM, Winbanks CE, Boey EJ, Tham YK, Kiriazis H et al. Therapeutic inhibition of the miR-34 family attenuates pathological cardiac remodeling and improves heart function. Proc Natl Acad Sci USA 2012; 109: 17615–17620. 23047694 10.1073/pnas.1206432109 PMC3491509

[bib20] Hullinger TG, Montgomery RL, Seto AG, Dickinson BA, Semus HM, Lynch JM et al. Inhibition of miR-15 protects against cardiac ischemic injury. Circ Res 2012; 110: 71–81. 22052914 10.1161/CIRCRESAHA.111.244442 PMC3354618

[bib21] Aurora AB, Mahmoud AI, Luo X, Johnson BA, van Rooij E, Matsuzaki S et al. MicroRNA-214 protects the mouse heart from ischemic injury by controlling Ca(2)(+) overload and cell death. J Clin Invest 2012; 122: 1222–1232. 22426211 10.1172/JCI59327 PMC3314458

[bib22] Li J, Li Y, Jiao J, Wang J, Li Y, Qin D et al. Mitofusin 1 is negatively regulated by microRNA 140 in cardiomyocyte apoptosis. Mol Cell Biol 2014; 34: 1788–1799. 24615014 10.1128/MCB.00774-13 PMC4019028

[bib23] Bang C, Batkai S, Dangwal S, Gupta SK, Foinquinos A, Holzmann A et al. Cardiac fibroblast-derived microRNA passenger strand-enriched exosomes mediate cardiomyocyte hypertrophy. J Clin Invest 2014; 124: 2136–2146. 24743145 10.1172/JCI70577 PMC4001534

[bib24] Ong SG, Lee WH, Huang M, Dey D, Kodo K, Sanchez-Freire V et al. Cross talk of combined gene and cell therapy in ischemic heart disease: role of exosomal microRNA transfer. Circulation 2014; 130: S60–S69. 25200057 10.1161/CIRCULATIONAHA.113.007917 PMC4862832

[bib25] Jansen F, Yang X, Hoelscher M, Cattelan A, Schmitz T, Proebsting S et al. Endothelial microparticle-mediated transfer of MicroRNA-126 promotes vascular endothelial cell repair via SPRED1 and is abrogated in glucose-damaged endothelial microparticles. Circulation 2013; 128: 2026–2038. 24014835 10.1161/CIRCULATIONAHA.113.001720

[bib26] Chan JK, Blansit K, Kiet T, Sherman A, Wong G, Earle C et al. The inhibition of miR-21 promotes apoptosis and chemosensitivity in ovarian cancer. Gynecol Oncol 2014; 132: 739–744. 24472409 10.1016/j.ygyno.2014.01.034

[bib27] Liwak-Muir U, Dobson CC, Naing T, Wylie Q, Chehade L, Baird SD et al. ERK8 is a novel HuR kinase that regulates tumour suppressor PDCD4 through a miR-21 dependent mechanism. Oncotarget 2015; 7: 1439–5. 10.18632/oncotarget.6363 PMC4811471 26595526

[bib28] Chen MC, Chang JP, Liu WH, Yang CH, Chen CJ, Fang CY et al. Increased serum oxidative stress in patients with severe mitral regurgitation: a new finding and potential mechanism for atrial enlargement. Clin Biochem 2009; 42: 943–948. 19414007 10.1016/j.clinbiochem.2009.04.012

[bib29] Ahmed MI, Gladden JD, Litovsky SH, Lloyd SG, Gupta H, Inusah S et al. Increased oxidative stress and cardiomyocyte myofibrillar degeneration in patients with chronic isolated mitral regurgitation and ejection fraction >60%. J Am Coll Cardiol 2010; 55: 671–679. 20170794 10.1016/j.jacc.2009.08.074 PMC3092364

[bib30] Hare JM, Mangal B, Brown J, Fisher C Jr., Freudenberger R, Colucci WS et al. Impact of oxypurinol in patients with symptomatic heart failure. Results of the OPT-CHF study. J Am Coll Cardiol 2008; 51: 2301–2309. 18549913 10.1016/j.jacc.2008.01.068

[bib31] Sahoo S, Losordo DW. Exosomes and cardiac repair after myocardial infarction. Circ Res 2014; 114: 333–344. 24436429 10.1161/CIRCRESAHA.114.300639

[bib32] Liu X, Hall SR, Wang Z, Huang H, Ghanta S, Di Sante M et al. Rescue of neonatal cardiac dysfunction in mice by administration of cardiac progenitor cells in utero. Nat Commun 2015; 6: 8825. 26593099 10.1038/ncomms9825 PMC4673493

[bib33] Thery C. Cancer: diagnosis by extracellular vesicles. Nature 2015; 523: 161–162. 26106856 10.1038/nature14626

[bib34] Pashkow FJ. Oxidative stress and inflammation in heart disease: do antioxidants have a role in treatment and/or prevention? Int J Inflam 2011; 2011: 514623. 21860805 10.4061/2011/514623 PMC3157078

[bib35] Feng Y, Huang W, Wani M, Yu X, Ashraf M. Ischemic preconditioning potentiates the protective effect of stem cells through secretion of exosomes by targeting Mecp2 via miR-22. PloS One 2014; 9: e88685. 24558412 10.1371/journal.pone.0088685 PMC3928277

[bib36] Gupta S, Knowlton AA. HSP60 trafficking in adult cardiac myocytes: role of the exosomal pathway. Am J Physiol Heart Circ Physiol 2007; 292: H3052–H3056. 17307989 10.1152/ajpheart.01355.2006

[bib37] Webber J, Steadman R, Mason MD, Tabi Z, Clayton A. Cancer exosomes trigger fibroblast to myofibroblast differentiation. Cancer Res 2010; 70: 9621–9630. 21098712 10.1158/0008-5472.CAN-10-1722

[bib38] Kogure T, Yan IK, Lin WL, Patel T. Extracellular vesicle-mediated transfer of a novel long noncoding RNA TUC339: a mechanism of intercellular signaling in human hepatocellular cancer. Genes Cancer 2013; 4: 261–272. 24167654 10.1177/1947601913499020 PMC3807642

[bib39] Jonas S, Izaurralde E. Towards a molecular understanding of microRNA-mediated gene silencing. Nat Rev Genet 2015; 16: 421–433. 26077373 10.1038/nrg3965

[bib40] Ren XP, Wu J, Wang X, Sartor MA, Qian J, Jones K et al. MicroRNA-320 is involved in the regulation of cardiac ischemia/reperfusion injury by targeting heat-shock protein 20. Circulation 2009; 119: 2357–2366. 19380620 10.1161/CIRCULATIONAHA.108.814145 PMC2746735

[bib41] Li RC, Tao J, Guo YB, Wu HD, Liu RF, Bai Y et al. *In vivo* suppression of microRNA-24 prevents the transition toward decompensated hypertrophy in aortic-constricted mice. Circ Res 2013; 112: 601–605. 23307820 10.1161/CIRCRESAHA.112.300806 PMC3622206

[bib42] Cheng Y, Liu X, Zhang S, Lin Y, Yang J, Zhang C. MicroRNA-21 protects against the H(2)O(2)-induced injury on cardiac myocytes via its target gene PDCD4. J Mol Cell Cardiol 2009; 47: 5–14. 19336275 10.1016/j.yjmcc.2009.01.008 PMC3593965

[bib43] Wang JX, Zhang XJ, Li Q, Wang K, Wang Y, Jiao JQ et al. MicroRNA-103/107 regulate programmed necrosis and myocardial ischemia/reperfusion injury through targeting FADD. Circ Res 2015; 117: 352–363. 26038570 10.1161/CIRCRESAHA.117.305781

[bib44] Ibrahim AG, Cheng K, Marban E. Exosomes as critical agents of cardiac regeneration triggered by cell therapy. Stem Cell Rep 2014; 2: 606–619. 10.1016/j.stemcr.2014.04.006 PMC4050492 24936449

[bib45] Lin BR, Natarajan V. Negative regulation of human U6 snRNA promoter by p38 kinase through Oct-1. Gene 2012; 497: 200–207. 22310390 10.1016/j.gene.2012.01.041 PMC3306512

[bib46] Xu LF, Wu ZP, Chen Y, Zhu QS, Hamidi S, Navab R. MicroRNA-21 (miR-21) regulates cellular proliferation, invasion, migration, and apoptosis by targeting PTEN, RECK and Bcl-2 in lung squamous carcinoma. PloS One 2014; 9: e103698. 25084400 10.1371/journal.pone.0103698 PMC4118890

[bib47] Boelens MC, Wu TJ, Nabet BY, Xu B, Qiu Y, Yoon T et al. Exosome transfer from stromal to breast cancer cells regulates therapy resistance pathways. Cell 2014; 159: 499–513. 25417103 10.1016/j.cell.2014.09.051 PMC4283810

[bib48] Hu GW, Li Q, Niu X, Hu B, Liu J, Zhou SM et al. Exosomes secreted by human-induced pluripotent stem cell-derived mesenchymal stem cells attenuate limb ischemia by promoting angiogenesis in mice. Stem Cell Res Ther 2015; 6: 10. 26268554 10.1186/scrt546 PMC4533800

[bib49] Feng Y, Huang W, Meng W, Jegga AG, Wang Y, Cai W et al. Heat shock improves Sca-1+stem cell survival and directs ischemic Cardiomyocytes toward a prosurvival phenotype via exosomal transfer: a critical role for HSF1/miR-34a/HSP70 pathway. Stem Cells 2014; 32: 462–472. 24123326 10.1002/stem.1571 PMC5517317

