# Supplementary figures and images for: Cardiac progenitor cell-derived exosomes prevent cardiomyocytes apoptosis through exosomal miR-21 by targeting PDCD4
Source: Cell Death Dis. 2016 Jun 23;7(6):e2277–. doi: 10.1038/cddis.2016.181 (PMC5143405; doi:10.1038/cddis.2016.181)

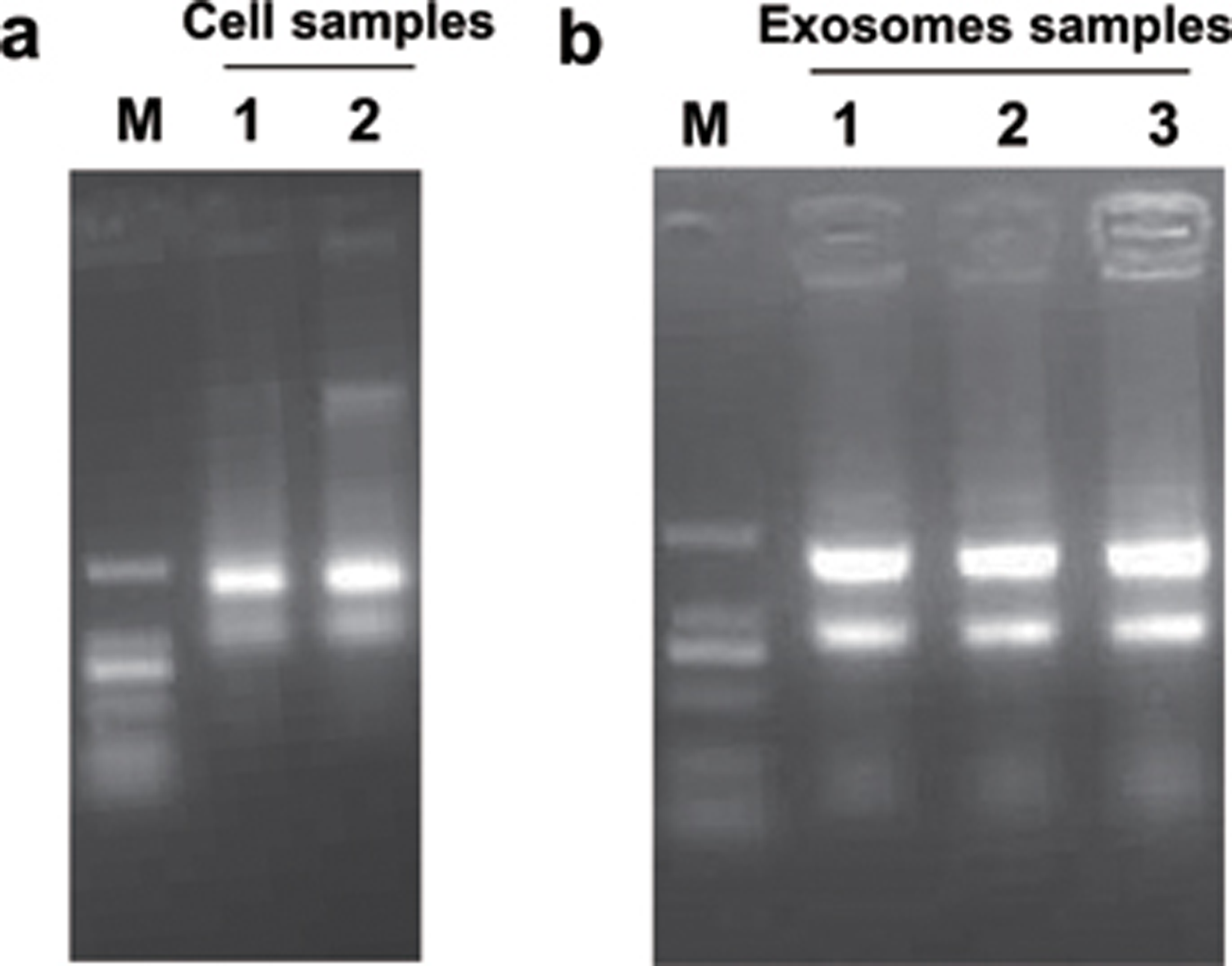

Supplement: Supplementary Figure S1 [file cddis2016181x1.tif]

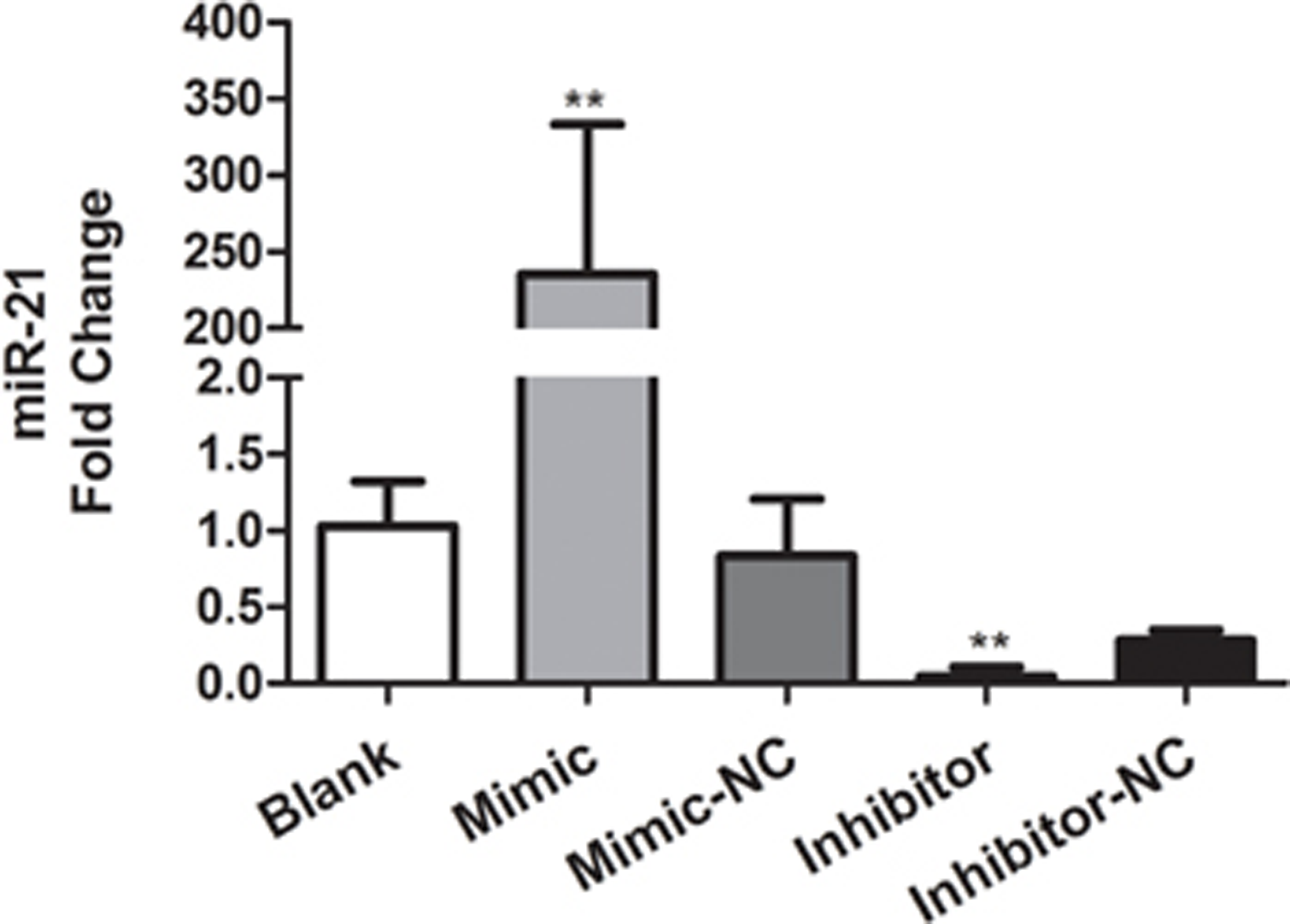

Supplement: Supplementary Figure S2 [file cddis2016181x2.tif]

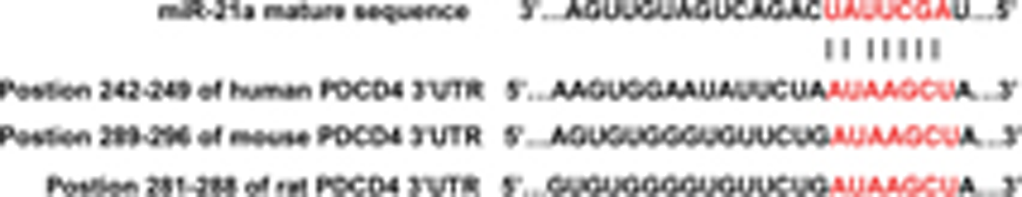

Supplement: Supplementary Figure S3 [file cddis2016181x3.tif]

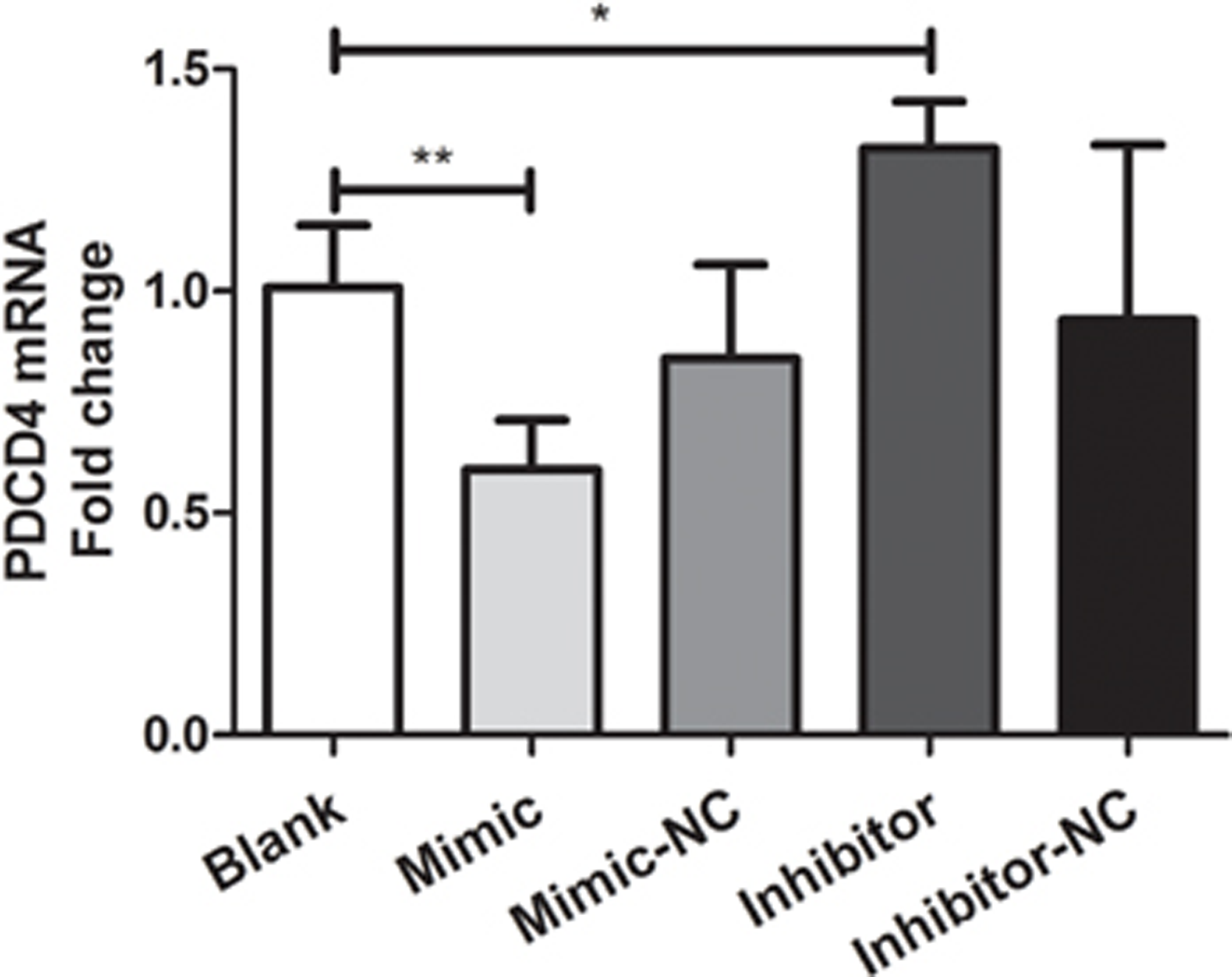

Supplement: Supplementary Figure S4 [file cddis2016181x4.tif]

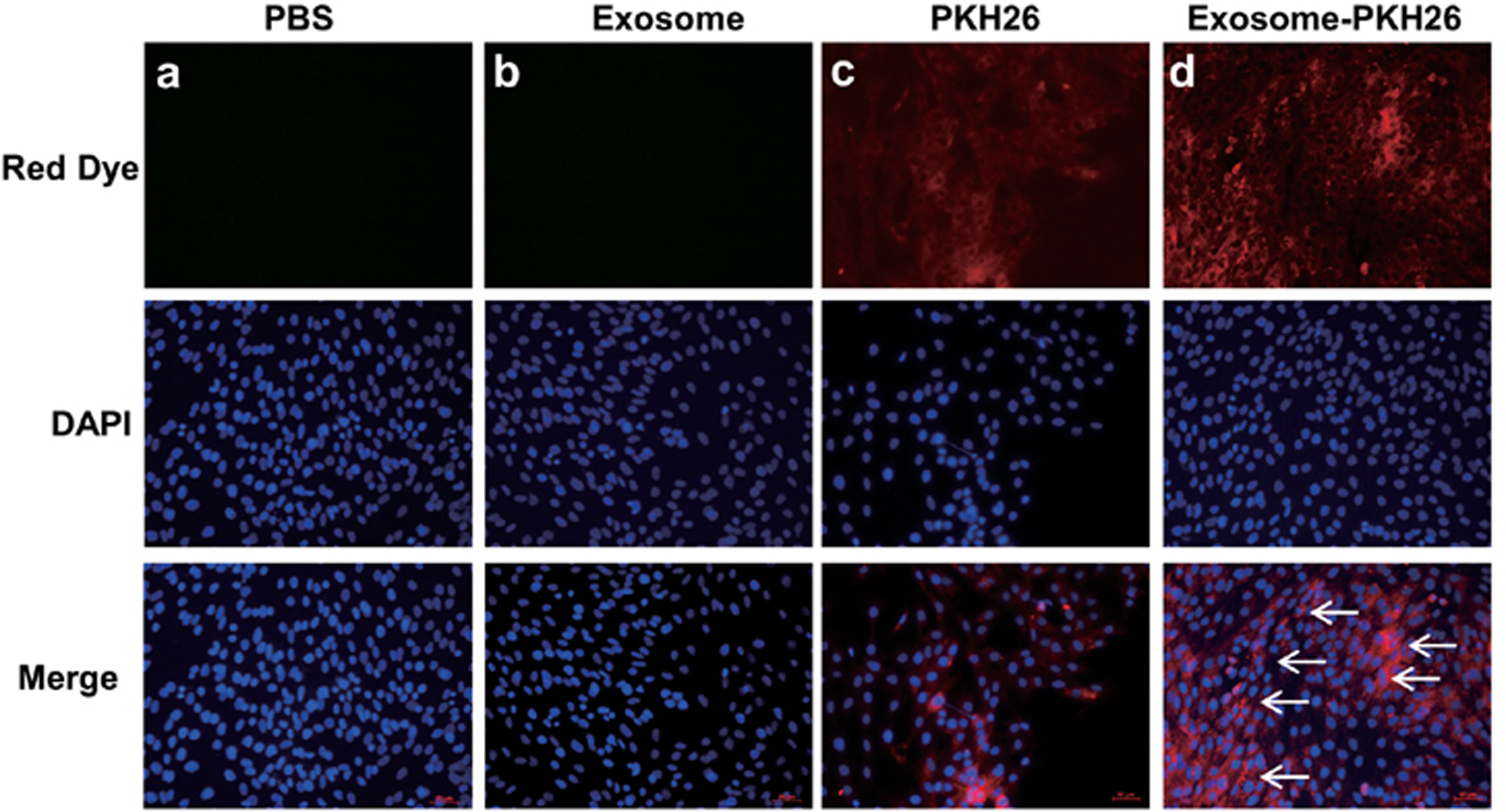

Supplement: Supplementary Figure S5 [file cddis2016181x5.tif]
