# Supplementary material for: Cardiac progenitor cell-derived exosomes prevent cardiomyocytes apoptosis through exosomal miR-21 by targeting PDCD4
Source: Cell Death Dis. 2016 Jun 23;7(6):e2277–. doi: 10.1038/cddis.2016.181 (PMC5143405; doi:10.1038/cddis.2016.181)
Supplement: Supplementary Figure Legends [file cddis2016181x6.docx]

**Supplementary Information**

**Figure S1** Image of agarose gel electrophoresis indicated cell (a) and exosomal (b) RNAs samples integrity.

**Figure S2** 100μM H_2_O_2_ treated H9C2 cells transfected with miR-21 mimics, inhibitors, and both negative controls, qPCR analyzed miR-21 levels in H9C2 cells (N=3, ***P*<0.01 vs blank group).

**Figure S3** Target scan predicted conserved binding sites in 3’UTR of PDCD4 mRNA in different species

**Figure S4** 100μM H_2_O_2_ treated H9C2 cells transfected with miR-21 mimics, inhibitors, and both negative controls, qPCR analyzed PDCD4 mRNA levels in H9C2 cells (N=3,**P*<0.05, ***P*<0.01 *vs* blank group).

**Figure S5** H9C2 cells internalization of CPC-derived exosomes.H9C2 cells were incubated with CPC-derived exosomes that were labeled with PKH26 (red) for 12 h. H9C2 cells were also incubated with PKH26 without exosomes, exosomes without PKH26 and PBS as negative controls to observe carryover of PKH26. Images of H9C2 cells incubated with negative controls are column (a-c), or PKH26 labeled exosomes are (d). The white arrow directed some PKH26 labeled exosomes pellets (red) showed in the cytoplasm of H9C2 cells, scale bar=50μM.
